# Decoding the Tissue-Specific Profiles of Bioactive Compounds in *Helvella leucopus* Using Combined Transcriptomic and Metabolomic Approaches

**DOI:** 10.3390/jof11030205

**Published:** 2025-03-06

**Authors:** Qian Zhou, Xusheng Gao, Junxia Ma, Haoran Zhao, Dan Gao, Huixin Zhao

**Affiliations:** 1 Xinjiang Key Laboratory of Special Species Conservation and Regulatory Biology, School of Life Sciences, Xinjiang Normal University, Urumqi 830054, China; zhouqian19900713@sina.com (Q.Z.); haoranzhao1999@126.com (H.Z.); 2 Key Laboratory of Biological Resources and Ecology of Pamirs Plateau in Xinjiang Uygur Autonomous Region, College of Life and Geography Sciences, Kashi University, Kashi 844000, China; gaoxusheng@o.cnu.ac.kr (X.G.); jxiama@163.com (J.M.); 3 Institute of Chinese Materia Medica, China Academy of Chinese Medical Sciences, Beijing 100700, China

**Keywords:** mushroom, tissue-specific metabolic, biosynthetic pathway network, nutritional components

## Abstract

*Helvella leucopus*, an endangered wild edible fungus, is renowned for its distinct health benefits and nutritional profile, with notable differences in the bioactive and nutritional properties between its cap and stipe. To investigate the molecular basis of these tissue-specific variations, we conducted integrative transcriptomic and metabolomic analyses. Metabolomic profiling showed that the cap is particularly rich in bioactive compounds, including sterols and alkaloids, while the stipe is abundant in essential nutrients, such as glycerophospholipids and amino acids. Transcriptomic analysis revealed a higher expression of genes involved in sterol biosynthesis (*ERG1*, *ERG3*, *ERG6*) and energy metabolism (*PGK1*, *ENO1*, *PYK1*) in the cap, suggesting a more active metabolic profile in this tissue. Pathway enrichment analysis highlighted tissue-specific metabolic pathways, including riboflavin metabolism, pantothenate and CoA biosynthesis, and terpenoid backbone biosynthesis, as key contributors to the unique functional properties of the cap and stipe. A detailed biosynthetic pathway network further illustrated how these pathways contribute to the production of crucial bioactive and nutritional compounds, such as sterols, alkaloids, linoleic acid derivatives, glycerophospholipids, and amino acids, in each tissue. These findings provide significant insights into the molecular mechanisms behind the health-promoting properties of the cap and the nutritional richness of the stipe, offering a theoretical foundation for utilizing *H. leucopus* in functional food development and broadening our understanding of bioactive and nutritional distribution in edible fungi.

## 1. Introduction

*Helvella leucopus*, commonly known as the white saddle mushroom or Bachu mushroom, is an edible and medicinal fungus found only in certain regions of Xinjiang, China [1,2]. It is renowned for its unique morphology and rich content of bioactive compounds. *H. leucopus* typically forms symbiotic relationships with oak, pine, and beech trees and thrives in moist forest soils rich in humus, which supports the growth of its distinctive cap and stem structures [3]. These structures contain a variety of bioactive compounds, including polysaccharides, phenolics, terpenes, and nitrogenous compounds, which may have antioxidant, anti-inflammatory, and immunomodulatory properties [4,5,6]. Given the growing interest in functional foods and natural products, understanding the chemical and molecular mechanisms underlying the production of these bioactive compounds is crucial.

In Xinjiang, people often use the cap of *H. leucopus* to make soup due to its unique flavor, discarding the stem. The morphological, textural, and chemical differences between the cap and stem are likely linked to their distinct ecological adaptations and metabolic needs [7]. However, despite the ecological and biological significance of these structures, systematic comparative research on the genetic expression and metabolic composition differences between them remains limited [8,9].

Existing research has begun to reveal the chemical diversity within *H. leucopus* [10], using comparative metabolomics to identify significant differences in the distribution of polysaccharides, phenolics, and nitrogen-containing metabolites between the cap and stem [11]. These differences in bioactive compound distribution are likely related to the distinct functional roles of the two structures. Additionally, the importance of these compounds in *H. leucopus*’s antioxidant and anti-inflammatory activities has been emphasized, which could be valuable for preventing diseases associated with oxidative stress [12,13,14].

Despite these findings, several challenges remain in understanding the bioactive properties of *H. leucopus*. First, comprehensively understanding the molecular pathways governing the synthesis and regulation of bioactive compounds in the cap and stem is complex. Second, there is a need to investigate the relationship between specific genes and the biosynthesis of these compounds, which may vary significantly across tissues. Additionally, environmental factors such as soil composition, symbiotic relationships, and seasonal changes may influence the production of these compounds, necessitating further exploration.

Recent advances in omics technologies, particularly transcriptomics and metabolomics, have significantly enhanced the understanding of fungal metabolic pathways [15,16]. Transcriptomics provides insights into the dynamic regulation of gene networks, especially those involved in secondary metabolite synthesis [17,18]. Metabolomics, on the other hand, measures the types and quantities of metabolites, providing a snapshot of the biochemical processes in fungi [19,20]. For instance, omics technologies have revealed the biosynthetic pathways of polysaccharides and triterpenoids in *Ganoderma lucidum*. Similarly, transcriptomic and metabolomic analyses have elucidated the synthesis and regulation mechanisms of secondary metabolites in *Cordyceps sinensis* [21]. These examples demonstrate the critical role of omics technologies in deciphering fungal metabolic pathways and bioactive compound synthesis [22,23].

This study aims to apply integrated transcriptomics and metabolomics techniques to analyze the genetic expression and metabolic differences between the cap and stem of *H. leucopus*, revealing the composition of their bioactive compounds and potential molecular mechanisms. Initially, performing transcriptomic profiling on the cap and stem of *H. leucopus* may lead to their identification. Meanwhile, metabolomic analyses may characterize the differential distribution of key metabolites in those parts of this species. By combining these data, the mechanisms underlying bioactive compound synthesis and regulation can be clarified. Additionally, exploring the potential biological functions of these compounds through a comprehensive literature review will provide new scientific insights into the sustainable utilization and in-depth research of *H. leucopus*.

## 2. Materials and Methods

### 2.1. Materials and Chemicals

Samples were collected from Bachu County, Kashgar Prefecture, Xinjiang Uygur Autonomous Region, China (39.7831° N, 78.5492° E, altitude 1234 m). The average annual temperature in this area is 11.8 °C, with annual precipitation ranging between 50 and 100 mm.

Twelve experimental samples were collected from the fruiting bodies of the mushroom and were divided into *Helvella leucopus* pileus (HLP) and *Helvella leucopus* stipes (HLS). Immediately after collection, these samples were preserved in liquid nitrogen and then stored in a −80 °C refrigerator at Xinjiang Normal University until needed for metabolomics and transcriptomics analyses. All chemicals utilized in this study were chromatographic grade for liquid chromatography (LC). Acetonitrile, methanol, and formic acid were sourced from Merck (Darmstadt, Germany), while acetic acid and methanol were obtained from Tedia (Fairfield, OH, USA). Deionized water was purified using a Milli-Q system from Millipore (Billerica, MA, USA).

### 2.2. Scanning Structure and Ultrastructural Spectrometry Analysis

To investigate the microstructural and ultrastructural characteristics of *H. leucopus*, representative samples were selected from both the cap and stem. High-resolution scanning electron microscopy (SEM) was employed to analyze the surface morphology and structural details at the microscopic scale. Before imaging, the sample surfaces were meticulously cleaned using either anhydrous ethanol or ultrapure water to remove any debris or impurities that could interfere with imaging. To prevent deformation or damage caused by residual solvent in the vacuum environment, the cleaned samples were thoroughly dried in a ventilated oven. Finally, a 60-s gold sputtering process was applied to enhance conductivity and improve image quality for spectrometric analysis.

### 2.3. Determination of Nutrient Composition

In this study, several parameters were analyzed, including triterpenes, sterols, crude protein, total phenols, crude polysaccharides, and total flavonoids [24]. Total polysaccharides were quantified using the phenol-sulfuric acid method, while total flavonoids and phenols were determined using the aluminum nitrate and Folin-Ciocalteu colorimetric methods, respectively. The quantification of total triterpenoids was performed using the vanillin-perchloric acid method, following the standards T/AHFIA 004-2018 [25], SN/T 4260-2015 [26], SN/T 4592-2016 [27], T/AHFIA 005-2018 [28], and NY/T 3676-2020 [29]. The sulfate–phosphate–ferric method was used to measure total sterols, and crude protein content was assessed with a BCA kit (Pierce, Rockford, IL, USA) according to the manufacturer’s guidelines.

### 2.4. Transcriptomic Analysis

Total RNA was isolated from *H. leucopus* samples using the TRIzol kit (Santa Clara, CA, USA), following the manufacturer’s protocol. RNA integrity was examined with an Agilent 2100 bioanalyzer (Santa Clara, CA, USA) and validated through RNase-free agarose gel electrophoresis. To conduct transcriptomic analysis of different *H. leucopus* parts, six cDNA libraries were constructed using the Hieff NGS Ultima Dual-mode mRNA Library Prep Kit (Shanghai, China), with each library containing three biological replicates from pileus and stipes. Library quality assessment was performed using a high-sensitivity DNA assay kit (Santa Clara, CA, USA). High-throughput sequencing was carried out on the Illumina NovaSeq 6000 platform by Gene Denovo (Guangzhou, China). Low-quality reads were removed using the Fastp tool (version 0.18.0) to yield high-quality clean reads, which were subsequently assembled de novo with Trinity (version 2.11.0). The resulting transcripts were annotated through BLASTx (E-value < 1 × 10^−5^) against databases, including NCBI Non-Redundant Protein (Nr), Swiss-Prot, Pfam, KEGG, GO, and COG/KOG. Gene expression levels were estimated in TPM (transcripts per kilobase million) via RSEM V.1.1.17 and differentially expressed genes (DEGs) between parts were identified with edgeR (FDR < 0.05 and |log2Fold Change| > 1). Sequences of DEGs sequences were matched with the PlantTFDB database for transcription factor (TF) family classification, using *Arabidopsis thaliana* as a reference, with results confirmed against the TAIR database.

### 2.5. Metabolomic Analysis

The sample was dissolved in 500 μL of 80% methanol in water. A portion of the supernatant was then diluted with LC-MS-grade water to adjust the methanol concentration to 53%. Prior to analysis, samples were thoroughly mixed, and an equal volume from each was pooled to create a quality control sample. Metabolomic analysis was performed using high-performance liquid chromatography-tandem mass spectrometry (LC-MS/MS) at Gene Denovo (Guangzhou, China), with three replicates per sample group. Instrument parameters are detailed in Supplementary Table S1. Samples were analyzed in multiple reaction monitoring modes, utilizing the Gene Denovo database for accurate qualitative and quantitative assessment. SCIEX software (version 14.0) was used for chromatographic peak integration and correction, with relative compound concentrations calculated based on peak areas.

### 2.6. Statistical Analysis

Statistical analysis was conducted using SPSS 25.0.0.2(Armonk, NY, USA), applying one-way ANOVA to compare differences between sample groups. Significant differences were identified using the least significant difference method at *p* < 0.05. Data visualization was performed in GraphPad Prism 9.0 (La Jolla, CA, USA). Metabolic heat maps based on correlation were generated with Tbtools 1.047, and circle and bubble charts were created using the OmicShare online tool (https://www.omicshare.com/tools/, accessed on 7 October 2024) to enhance data visualization and interpretation.

## 3. Results

### 3.1. Changes in Morphological and Bioactive Nutrient of H. leucopus at Stalks and Pileus

**Figure 1:**
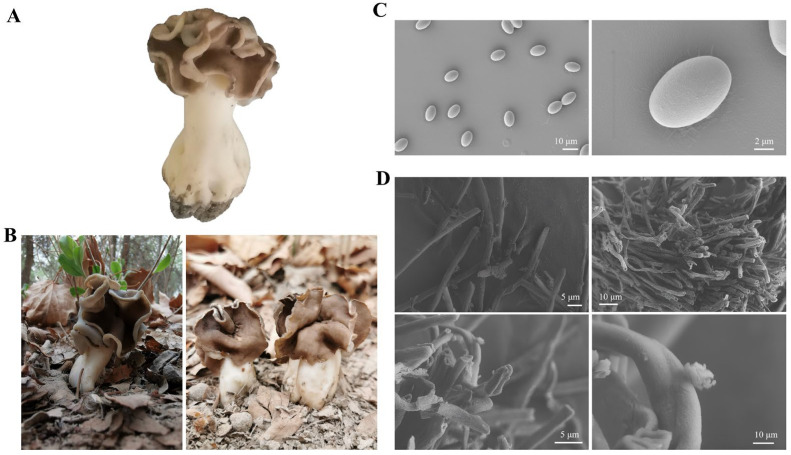
Comparison of morphology, sampling sites, spore structure, and nutritional components of *Helvella leucopus*. (**A**) Natural growth state of *H. leucopus*; (**B**) The habitat map of *H. leucopus*; (**C**) Spore images of *H. leucopus* under a scanning electron microscope; (**D**) Mycelium images of *H. leucopus* under a scanning electron microscope. The four images are each shown under different magnifications.

**Figure 2:**
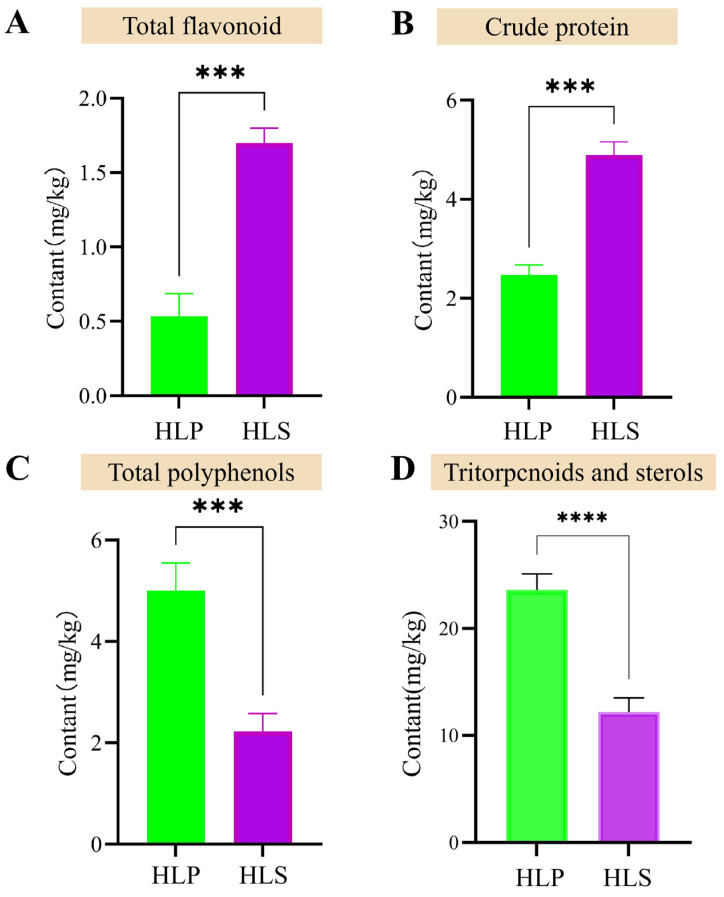
Comparison of bioactive components content in mushrooms from different groups (HLP and HLS). (**A**) The content of total flavonoids; (**B**) The content of crude protein, triterpenes, and sterols; (**C**) The content of total polyphenols; (**D**) The content of triterpenes and sterols. Data are presented as mean ± standard deviation, with significant differences determined through statistical analysis (*** *p* < 0.001, **** *p* < 0.0001).

The phenotypic images of *H. leucopus* reveal significant color differences between the stipe and the cap (Figure 1A). Scanning electron microscopy (SEM) images of the spores from the cap show that the spores are oval or spindle-shaped with smooth surfaces and no distinct texture. The spores measure between 15–22 μm in length and 10–15 μm in width, with thin spore walls, a simple structure, and no notable pores (Figure 1C,D). Further analysis of the nutrient and active ingredient contents revealed that the stipe has higher levels of total protein and total flavonoids, with significant differences observed. Additionally, we found that the cap contains higher levels of total polyphenols, triterpenoids, and sterols (Figure 2).

### 3.2. Metabolomics Analysis of the Stipe and Cap of H. leucopus

To further examine the metabolic differences between the pileus and stems of *H. leucopus*, the metabolic profiles of samples HLP and HLS were analyzed using ultra-performance liquid chromatography-tandem mass spectrometry (UPLC-MS/MS). The stability of the UPLC-MS/MS system was initially evaluated by comparing the base peak plots of the mass spectra from quality control (QC) samples with a principal component analysis (PCA) of all samples. Consistency in base peaks across positive and negative ion detection modes, in terms of response intensity and retention times, indicated minimal variation from instrumental errors, thereby confirming high data reliability (Figure 2A,B).

**Figure 3:**
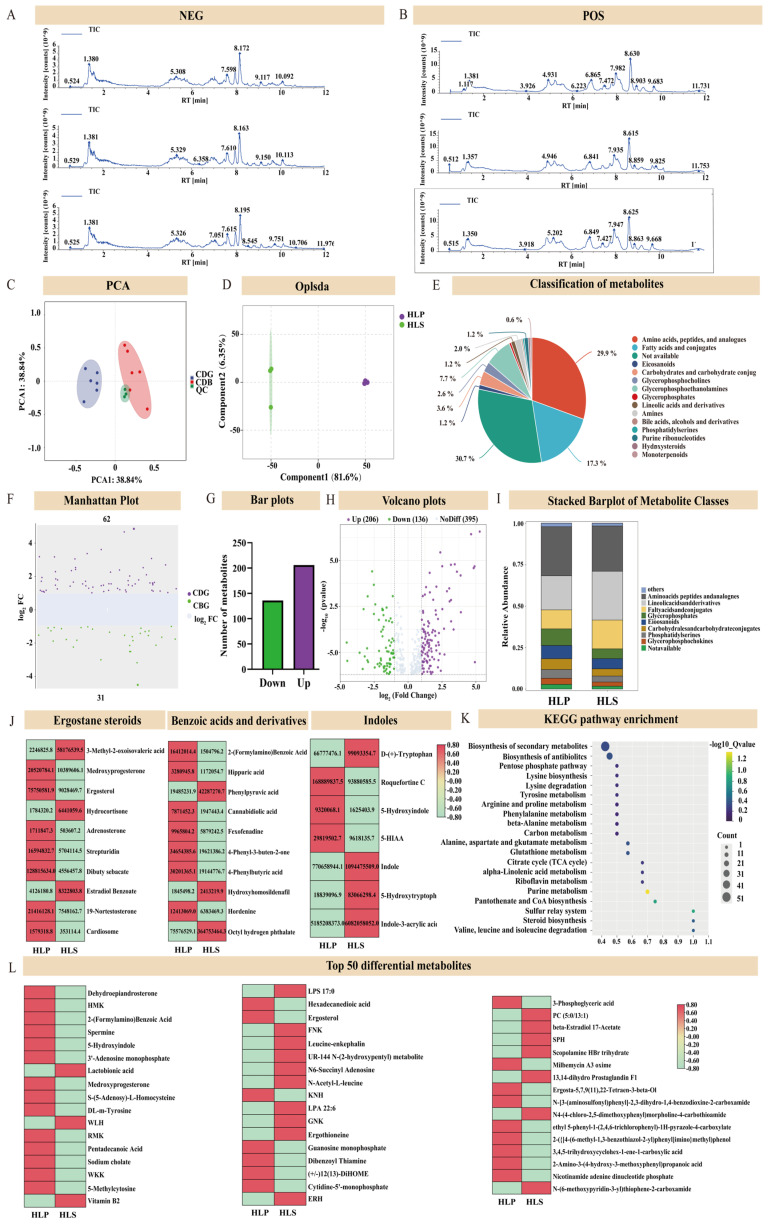
Metabolite differences in different nutritional parts of *Helvella leucopus*. (**A**) Total ion chromatogram (TIC) in negative ion mode (NEG). Subplots represent the results of three replicate experiments; (**B**) Total ion chromatogram (TIC) in positive ion mode (POS). Subplots represent the results of three replicate experiments; (**C**) Principal component analysis (PCA) plot showing the distribution of samples from different groups (HLP, HLS, QC); (**D**) Principal component distribution plot based on PLS-DA analysis; (**E**) Pie chart of the relative percentage content of different compound classes; (**F**) Volcano plot of differential metabolites; (**G**) Bar chart showing the number of upregulated and downregulated metabolites; (**H**) Scatter plot of log2 fold changes and -log10 *p*-values for differential metabolites; (**I**) Stacked bar chart of the relative abundance of various compound classes; (**J**) Heatmap of the relative content of three metabolites; (**K**) Analysis of the top 20 different metabolic pathways for differential metabolites; (**L**) KEGG enrichment analysis plot for the top 50 differential metabolites.

Preliminary studies have shown that the pileus of *H. leucopus* is significantly different from the stalks in terms of sensory and flavor, and in order to further explore the reasons for this, we conducted a metabolomics study based on UPLC-Q-TOF/MS. PCA revealed significant differences in metabolite composition among the samples within each cluster, including six biological replicates (Figure 3C). This result was further validated by OPLS-DA (Figure 3D). These metabolites were systematically categorized into 13 different classes, including Carboxylic acids and derivatives (36), Carboxylic acids and derivatives (28), Organooxygen compounds (16), Benzenoids (10), Steroids and steroid derivatives (9), Indoles and derivatives (7), Organonitrogen compounds (5), Purine nucleosides (5), Keto acids and derivatives (4), and so on (Figure 3E). Detailed information on these metabolites can be found in Supplementary Table S2. The differences in compound levels between the two groups were visualized using stacked bar charts.

### 3.3. Differential Analysis of the Stipe and Cap of H. leucopus

To explore the metabolic differences between the pileus and stems of *H. leucopus*, Variable Importance in Projection (VIP) analysis was conducted using the OPLS-DA algorithm. VIP scores highlight the impact of each metabolite on the classification differences between the two groups, with a VIP score ≥1 indicating a significant difference. Metabolites with fold change (FC) values ≥2 and *p* ≤ 0.05 were initially screened, identifying notable expression differences between HLP and HLS groups. Through qualitative and quantitative analysis of the detected metabolites, the researchers compared FC values between the groups. After log-transforming the FC values, 179 differential metabolites were identified, with 107 showing upregulation and 72 showing downregulation (Figure 3F–H). It is evident that the cap (HLP) contains higher levels of fatty acids compared to the stem (HLS). Additionally, the cap exhibits higher concentrations of glycerophospholipids, steroids, and steroid derivatives (Figure 3I).

This indicates that the metabolism in HLP is more active than in HLS. Steroidal compounds exhibit significant pharmacological activities, such as anti-tumor and anti-inflammatory effects [30,31]. In this study, a total of 10 different steroidal compounds were identified. Among these, Dibutyl sebacate had the highest content. Additionally, seven compounds had the highest concentrations in HLS, including Medroxyprogesterone, Ergosterol, Hydrocortisone, Adrenosterone, Strophanthidin, Dibutyl sebacate, and Cortodoxone. The remaining compounds had higher concentrations in HLP. Additionally, studies have shown that the polyphenolic compounds in mushrooms offer various health benefits, including antioxidant, anti-inflammatory, antibacterial, anticancer, lipid and glucose regulation, and neuroprotective effects. In this study, a total of seven different polyphenolic compounds were identified. Four of these compounds were highly expressed in HLP, namely tryptophan, Indole, 5-Hydroxytryptophan, and Indole-3-acrylic acid. Benzoic acid and its derivatives found in mushrooms are widely used in food preservation, drug development, anti-inflammatory and analgesic treatments, as well as antibacterial and anticancer formulations. In this study, 10 benzoic acids and their derivatives were identified, most of which had higher concentrations in HLP (Figure 3J). Additionally, we ranked the metabolites by *p*-value and displayed the top 50. The results indicate that the metabolism in HLP is more active (Figure 3L).

In this study, we mapped the differential metabolites (DEMs) to KEGG pathways, identifying over 30 pathways in total. Key pathways included terpenoid backbone biosynthesis, secondary metabolite biosynthesis, amino acid biosynthesis, lysine biosynthesis, and lysine degradation. These results offer valuable insights into the metabolomic distinctions between different tissue parts (Figure 3K).

### 3.4. Transcriptomics Profilin of the Stipe and Cap of H. leucopus

In the present investigation, we examined pre- and post-excavation *H. leucopus* specimens that were analyzed by RNA-seq. A total of 10 cDNA libraries were obtained for HLP and HLS specimens. After excluding low-quality data, a total of 18,983 valid reads with 66.46 G valid bases were obtained, as shown in Table S4. The Q20 and Q30 values of all samples were above 96.1% and 91.2%, respectively, and the GC content was between 52.5%~52.73% (Table S3). This analysis yielded a total of 18,983 annotated genes. Among these, 8702 genes (45.84% of the total) were annotated by the NCBI-NR database. Additionally, 4978 by the GO database, and 7663 by the KEGG database (Table S4).

**Figure 4:**
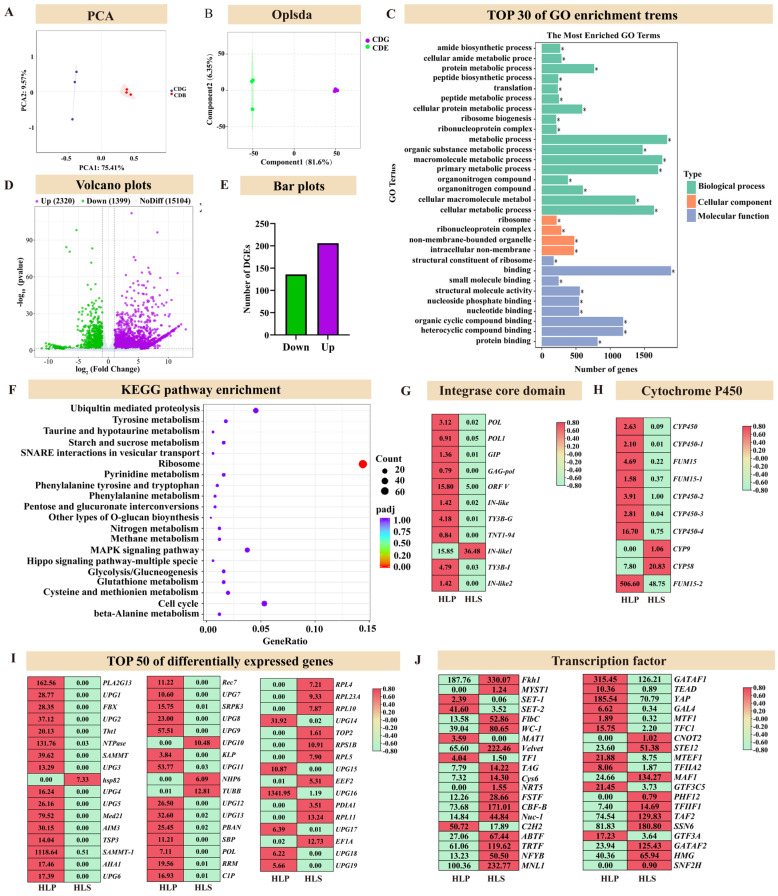
Transcriptomic differences in different nutritional parts of *Helvella leucopus*. (**A**) Principal component analysis (PCA) plot of different nutritional parts; (**B**) Principal component analysis (PCA) plot of different nutritional parts; (**C**) GO enrichment analysis plot of differentially expressed genes (* *p* < 0.05); (**D**) Volcano plot showing the significance and fold changes of gene expression differences between sample groups. Green dots represent downregulated genes, and purple dots represent upregulated genes; (**E**) Bar chart showing the number of upregulated and downregulated genes in different sample groups (HLS and HLP); (**F**) Dot plot showing the significance (padj) and gene ratio (GeneRatio) of different metabolic pathways in the samples; (**G**) Heatmap showing the expression of integrase core domain-related genes in different sample groups (HLS and HLP); (**H**) Heatmap showing the expression of Cytochrome P450-related genes in different sample groups (HLS and HLP); (**I**) Heatmap showing the expression of the top 50 differentially expressed genes in different sample groups (HLS and HLP); (**J**) Heatmap showing the expression of the differentially expressed transcription factor genes in different sample groups (HLS and HLP).

The PCA of the identified transcripts from the two experimental samples demonstrated distinct differences in gene expression levels among the three biological replicates for each treatment (Figure 4A). Differential gene analysis, applying the criteria of |log2Fold Change| ≥ 1 and FDR < 0.05, identified 3719 differentially expressed genes (DEGs), with 2320 upregulated and 1399 downregulated. These DEGs were visualized through volcano plots and bar charts to provide a clearer depiction of expression patterns (Figure 4C,D).

The DEGs identified in this study were annotated using KEGG and GO public databases. GO enrichment analysis highlighted a significant enrichment of DEGs across the categories of biological processes, cellular components, and molecular functions. The results showed notable enrichment of genes associated with metabolism, synthesis, transport, and binding functions, especially within protein and peptide metabolic processes and ribosome-related functions (Figure 3E and Supplementary Table S6).

KEGG annotation revealed that DEGs were involved in multiple biological pathways, including environmental information processing, genetic information processing, organismal systems, and metabolism. Significant enrichment was observed in pathways related to ubiquitin-mediated proteolysis, tyrosine metabolism, starch and sucrose metabolism, SNARE interactions in vesicular transport, ribosome function, purine metabolism, phenylalanine metabolism, glutathione metabolism, and the cell cycle (Figure 4F). In this figure, the dot size represents the number of genes within each pathway, while the color indicates the significance level. These results highlight substantial differences in gene expression across various biological processes.

In fungi, the integrase core domain genes are involved in processes such as DNA recombination, integration, and transposition, aiding fungi in adapting to changing environmental conditions. These genes are essential for the growth, development, and environmental adaptability of fungi. In this study, most of these genes were highly expressed in HLP, consistent with the metabolomic data, indicating that HLP exhibits a more active metabolism (Figure 4G,H).

In mushrooms, transcription factors play a crucial role in growth, development, metabolism, and environmental adaptation by regulating gene expression. The functions of these transcription factors ensure the normal growth and adaptability of mushrooms under various environmental conditions. Interestingly, these transcription factors are all highly expressed in HLS.

### 3.5. Integrated Analysis of Metabolomics and Transcriptomics

**Figure 5:**
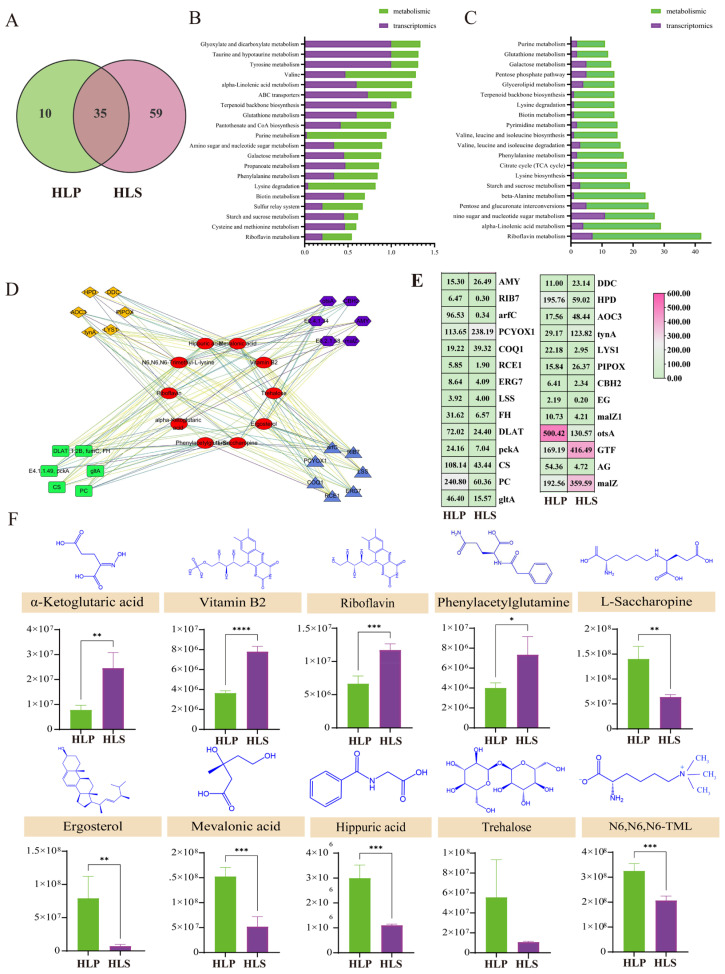
Multi-omics analysis of metabolites in different nutritional parts of *H. leucopus*. (**A**) Venn diagram of significantly differentially expressed metabolites in metabolomics (green) and transcriptomics (purple); (**B**) Number of metabolites and transcripts in different metabolic pathways; (**C**) Cumulative number of significantly changed metabolites and genes in different metabolic pathways; (**D**) Metabolite-gene network pathway diagram showing the interactions between metabolites (green and yellow diamonds), genes (blue triangles), and proteins (red circles); (**E**) Relative abundance of significantly changed metabolites; (**F**) Bar chart of the relative abundance of some significantly changed metabolites, with data presented as mean ± standard deviation. Statistical significance is indicated as follows: * *p* < 0.05, ** *p* < 0.01, *** *p* < 0.001, **** *p* < 0.0001.

To further explore the role of gene expression in *H. leucopus* substance synthesis, this study systematically investigated the relationship between the cap and stipe DEMs and DEGs of *H. leucopus* by combining transcriptome and metabolome analysis. The KEGG pathways of the transcriptome and metabolome were compared, and the number of KEGG pathways involved in the transcriptome and metabolome are shown in Figure 4A. The overlapping area in the circles represents the number of KEGG pathways involved in the differentially accumulated metabolites and differentially expressed genes, as identified by both omics (Tables S7 and S8). There are 35 common pathways between the transcriptomics and metabolomics, including riboflavion metabolism, starch and sucrose metabolism, lysine biosynthesis, pantothenate and CoA biosynthesis, and terpenoid backbone biosynthesis, among others. Further analysis of the 35 common pathways revealed the top 20 KEGG pathways ranked by *p*-value, as shown in Figure 5C. Additionally, the top 20 KEGG pathways with the highest number of genes and metabolites were identified (Figure 4B). From bottom to top, the higher the bar, the more active the biological pathway in the tested samples. These integrated omics analyses indicate that these metabolites and genes are primarily involved in primary and secondary metabolism. In recent years, secondary metabolism has been proposed to play a crucial role in plant growth and development [32]. The common pathways shown in Figure 5B,C, such as terpenoid backbone biosynthesis, are the main pathways of secondary metabolism. To better understand the relationship between metabolomics and transcriptomics, we also performed network analysis and visualization of the differentially accumulated metabolites and genes enriched in the KEGG pathways using Cytoscape 3.10.1 As shown in Figure 5D–F, alpha-ketoglutaric acid, vitamin B2, riboflavin, ergostrol, and ctrehalose not only had the lowest *p*-value s but also directly participated in the riboflavin metabolism and terpenoid backbone biosynthesis pathways. A single metabolite can bind to multiple genes, and a single gene can bind to multiple metabolites when involved in a pathway. In summary, different parts of *H. leucopus* significantly affect the starch and sucrose metabolism, lysine biosynthesis, pantothenate and CoA biosynthesis, terpenoid backbone biosynthesis, and cirtrate cycle (TCA cycle).

## 4. Discussion

**Figure 6:**
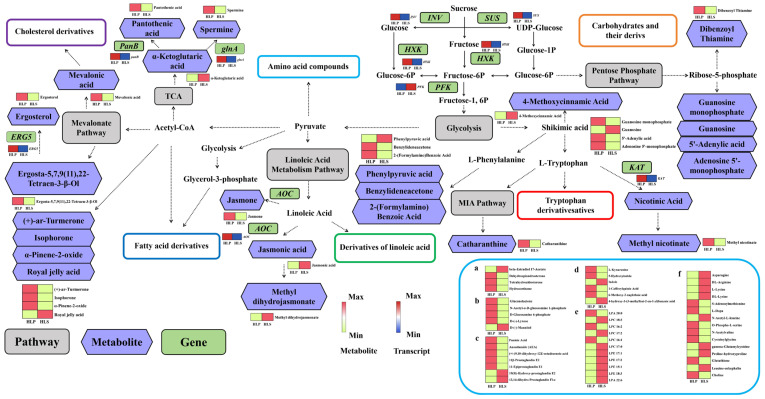
Visualization of metabolites and transcripts abundance in a biochemical network pathway map. The heatmap was plotted using log2-transformed values from metabolomic and transcriptome datasets; the cells in the heatmap from left to right represented HLP and HLS samples, respectively. TCA: tricarboxylic acid cycle; MIA: monoterpene indole alkaloid; *INV*: invertase gene; *SUS*: sucrose synthase gene; *HXK*: hexokinase gene; *PFK*: 6-phosphofructokinase gene; *AOC*: Allene oxide cyclase gene; *ERG5*: phosphomevalonate kinase gene; *PanB*: Pantothenate Synthetase gene; *glnA*: Glutamine Synthetase gene; *CCD*: Kynurenine Aminotransferase gene; a: Cholesterol derivatives; b: Carbohydrates and their derivatives; c: Derivatives of linoleic acid; d: Tryptophan derivatives; e: Fatty acid derivatives; f: Amino acid compounds.

Through integrated metabolomic and transcriptomic analyses, this study constructed a metabolic network map showcasing the biochemical differences between the cap and stipe tissues of *H*. *leucopus* (Figure 6). Functional differentiation during development has resulted in significant variations in gene expression and metabolite content between the cap and stipe.

### 4.1. Sterol Synthesis and Defense Mechanism

In this study, we found that *H. leucopus*’s cap exhibits significantly higher sterol synthesis activity compared to the stipe. The sterol compounds synthesized via the Mevalonate pathway and terpenoid backbone biosynthesis pathway, such as ergosterol and isophorone, play important roles in the biological activity of *H. leucopus*. Ergosterol is not only a crucial component of the cell membrane but also plays a vital role in stabilizing the membrane and enhancing the cell’s antioxidative capacity [33]. It is important for maintaining the fluidity and structural integrity of the membrane and also increases *H. leucopus*’s tolerance to oxidative stress, helping it adapt to various environmental stresses, such as UV radiation and pathogen invasion [24].

In addition, isophorone, a volatile metabolite synthesized through the Mevalonate pathway, has antioxidant, anti-inflammatory, and immunomodulatory properties [34]. Studies have shown that isophorone’s synthesis is closely associated with the organism’s defense mechanisms. It helps *H. leucopus* combat both biotic and abiotic environmental stresses by regulating antioxidative and immune responses. The accumulation of isophorone in the cap may enhance its resistance to pathogens and physical damage, further supporting the cap’s key role in defense and reproduction.

### 4.2. Fatty Acid Metabolism and Immune Regulation

In addition to sterol synthesis, significant differences in fatty acid metabolism were observed between the cap and stipe. The cap synthesizes jasmonic acid and other fatty acid derivatives through the linoleic acid metabolism pathway, which plays an important role in immune regulation in both plants and fungi [35,36]. Jasmonic acid, as a plant hormone, is known for its antioxidant, anti-inflammatory, and immune-enhancing functions. In this study, the cap exhibited significantly higher levels of jasmonic acid than the stipe, indicating that the cap may increase jasmonic acid synthesis to enhance its resistance to environmental stress and pathogens.

The synthesis of jasmonic acid is closely linked to the expression of the AOC gene, which encodes allene oxide cyclase [37]. The high expression of this gene in the cap reflects the cap’s heightened activity in response to external stress. Additionally, jasmonic acid and its derivatives regulate immune responses, increasing the survival capacity of *H. leucopus*, especially in the face of biotic and abiotic stresses.

### 4.3. Amino Acid Metabolism and Cell Growth

In contrast to the cap’s immune-regulatory metabolism, the stipe focuses primarily on cell growth and energy supply. We found that the amino acid biosynthesis pathway and the TCA cycle were significantly more active in the stipe, reflecting its central role in supporting the growth of *H. leucopus*. Amino acids are the foundation of protein synthesis and are essential for cell growth and division. In the stipe, the synthesis of phenylalanine and tryptophan was more pronounced, indicating that the stipe synthesizes these amino acids to provide the necessary structural building blocks for *H. leucopus* development. Phenylalanine is not only a precursor for synthesizing aromatic amino acids but also participates in the production of secondary metabolites, including plant hormones and anthocyanins, playing a significant biological role [38]. Tryptophan also plays a key role in cell growth, protein synthesis, and hormone regulation.

Meanwhile, the activity of the tricarboxylic acid cycle in the stipe underscores its critical role in energy supply. The TCA cycle generates ATP by converting carbohydrates, fatty acids, and amino acids, providing energy support for cell growth [39]. This ensures that *H. leucopus* can sustain cellular division and metabolism during growth. The high expression of key enzymes such as citrate synthase and malate dehydrogenase in the stipe indicates its reliance on the TCA cycle for energy production [40]. These metabolic activities ensure the proper growth of *H. leucopus*, providing the necessary energy and nutrients to support the development of the cap.

### 4.4. Tryptophan Metabolism and Growth Regulation

Tryptophan is a precursor for synthesizing various bioactive compounds, particularly plant hormones [41]. The metabolites derived from tryptophan, such as indole-3-acetic acid (IAA), are crucial for regulating cell division, elongation, and root development [42]. In *H. leucopus*, tryptophan metabolites such as IAA play a similar role in cell division and development, particularly in the growth of the stipe. In addition, tryptophan metabolism is not limited to hormone synthesis but also contributes to the production of nicotinic acid (vitamin B3). Nicotinic acid plays a vital role in cellular metabolism by promoting the synthesis of NAD+, which enhances cellular energy metabolism and supports cell growth and division [43]. In this study, both the cap and stipe of *H. leucopus* synthesized these important growth regulators, which are essential for maintaining normal cellular functions, promoting growth and development, and responding to environmental stress.

The integration of transcriptomics and metabolomics reveals the key roles of several genes in regulating metabolic pathways. For instance, the *Fkh1* gene plays an important role in regulating the cell cycle, while MYST1 and SET-1/2 genes influence gene expression through epigenetic modifications [44,45]. The WC-1 gene is involved in regulating light signal transduction, which is crucial for *H. leucopus* growth under varying light conditions [46]. Moreover, the Velvet complex controls the synthesis of secondary metabolites, which is consistent with the observed metabolic traits in the cap. The YAP and STE12 genes are crucial for cell proliferation and reproduction, respectively. Through the interplay of these genes and metabolic pathways, *H. leucopus* can adapt its metabolic network to various environmental conditions, ensuring proper growth and survival.

Although this study highlights the differences in metabolic pathways between the cap and stipe, the underlying regulatory mechanisms remain unclear. Future research can explore the role of transcription factors and key enzymes in regulating these metabolic pathways. Additionally, it will be important to study how environmental factors (such as temperature, humidity, and light) affect these metabolic pathways, especially in different growth stages. More detailed studies may uncover additional biological mechanisms, providing deeper insights into the biological functions of *H. leucopus* and supporting its applications in the food and pharmaceutical industries.

## 5. Conclusions

This study elucidated the biological basis for the functional differences between the cap and stipe of *H. leucopus* using integrated transcriptomic and metabolomic approaches. The cap was enriched with health-active components, such as sterols and alkaloids, while the stipe was rich in nutritional components, including glycerophospholipids and amino acids. Transcriptomic analysis revealed that genes involved in sterol synthesis and energy metabolism were highly expressed in the cap, indicating more active metabolic processes. The integrated omics analysis identified significant pathways, including riboflavin metabolism, pantothenate and CoA biosynthesis, and terpenoid backbone biosynthesis, which contribute to the functional differences between the cap and stipe. These findings offer crucial insights into the distinct health and nutritional values of different parts of *H. leucopus*, providing a theoretical foundation for its food development and further research on the functional differences in nutritional components within the fruiting body.

## Data Availability

The data used and or analyzed in this study have been uploaded to NCBI: PRJNA1178727.

## Supplementary Materials

The following supporting information can be downloaded at: https://www.mdpi.com/article/10.3390/jof11030205/s1, File S1. Supplement methods. Figure S1. Heat map of transcription factors. Table S1. Instrumental parameters. Table S2. Metabolite annotation results. Table S3. Sequencing Data Quality Statistics. Table S4. Annotated statistical tables. Table S5. Gene Expression and Functional Annotation Statistics. Table S6. GO enrichment analysis results. Table S7. KEGG enrichment analysis results. Table S8. KEGG enrichment analysis results.
