# Supplementary material for: Decoding the Tissue-Specific Profiles of Bioactive Compounds in Helvella leucopus Using Combined Transcriptomic and Metabolomic Approaches
Source: J Fungi (Basel). 2025 Mar 6;11(3):205. doi: 10.3390/jof11030205 (PMC11943342; doi:10.3390/jof11030205)
Supplement: Supplementary file 1 [file jof-11-00205-s001.zip › File S1. Supplement methods.pdf]

Determination of nutrient composition and micro- and macro-elements

The determination of polysaccharide content involves precise weighing of a 2 g sample powder, followed by reflux extraction in 100 mL of distilled water for 3 hours. The mixture is then hot filtered at a pressure of 0.07 ~ 0.08 MPa, with subsequent washing of the filter and residue using distilled water. This extraction process is repeated three times. The combined extracts are concentrated to 30 mL using a water bath and then mixed with 75 mL of anhydrous ethanol. After overnight incubation at 4 °C, the mixture is centrifuged at 2432 g for 15 minutes. The resulting precipitate is dissolved in distilled water and the volume is adjusted to 100 mL. The polysaccharide content is determined by measuring the absorbance at 490 nm using the phenol sulfuric acid method. A standard curve is constructed using glucose solutions with different mass concentrations to calculate the polysaccharide content in each sample.

For the determination of flavonoid content, a 2 g sample powder is finely weighed and then subjected to backflow extraction in 100 mL of 95% ethyl alcohol for 5 hours. The mixture is then filtered at a pressure of 0.07 ~ 0.08 MPa, followed by washing of the filter and residue. The extract is concentrated on a water bath to a volume of 5 ~ 10 mL and washed with 60% ethyl alcohol. The absorbance at 504 nm is measured using the nitrite-aluminum nitrate method, and the flavonoid content in each sample is calculated using standard curves constructed with rutin solutions of different mass concentrations.

The content of triterpenes and sterols is determined by ultrasonic extraction of 2 g of sample powder in a test tube with a plug using 30 mL of 75% ethanol for 3.5 hours at a power of 100 W and a frequency of 50 Hz. The mixture is then filtered at a pressure of 0.07 ~ 0.08 MPa, with subsequent washing of the filter and residue using 75% ethanol. The filtrate is transferred to a 50 mL volumetric bottle and the absorbance at 550 nm is measured using the vanilla-perchloric acid method. The contents of triterpenes and sterols in the samples are calculated using standard curves constructed with oleanolic acid solutions of different mass concentrations.

To determine the polyphenol content, a 2 g sample powder is accurately weighed and subjected to ultrasonic extraction in methanol for 3.5 hours at a power of 100 W and a frequency of 50 Hz. The extraction liquid is collected after filtration and the volume is adjusted to 50 mL. The absorbance at 765 nm is measured using the Folin phenol method. The polyphenol content in the sample is calculated using standard curves constructed with gallic acid solutions of different mass concentrations.

For the determination of crude protein content, a 0.1 g sample powder is accurately weighed and analyzed using an automatic Kjeldahl nitrogen analyzer. Similarly, the content of macro elements, trace elements, and heavy metals in the sample is determined by using an inductively coupled plasma mass spectrometer after accurately weighing a 0.1 g sample powder.
