# Supplementary figures and images for: Decoding the Tissue-Specific Profiles of Bioactive Compounds in Helvella leucopus Using Combined Transcriptomic and Metabolomic Approaches
Source: J Fungi (Basel). 2025 Mar 6;11(3):205. doi: 10.3390/jof11030205 (PMC11943342; doi:10.3390/jof11030205)

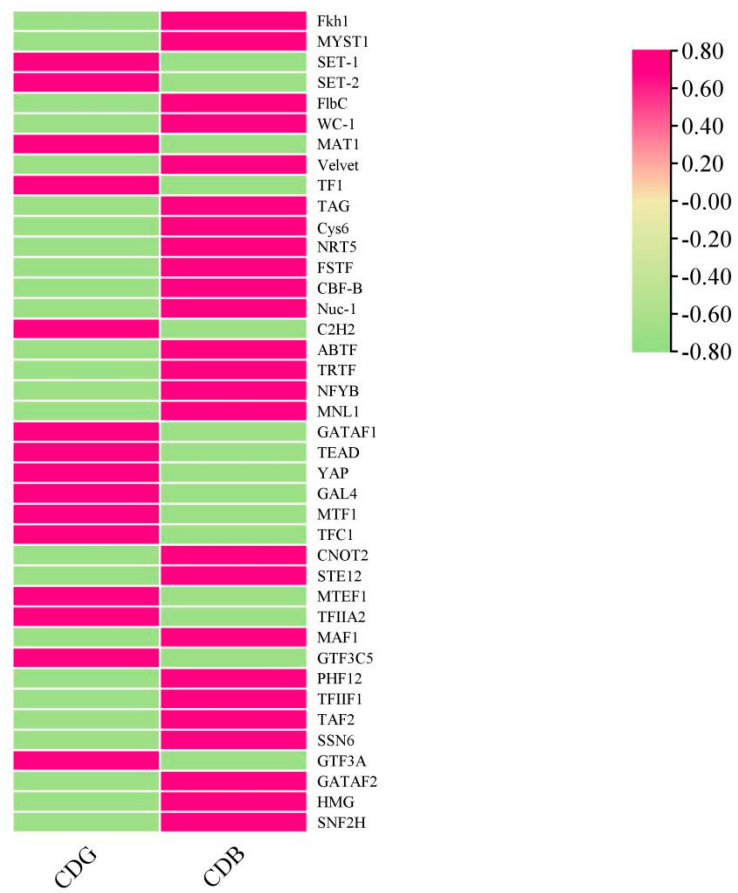

**Figure S1.** Heat map of transcription factors

Supplement: Supplementary file 1 [file jof-11-00205-s001.zip › Figure S1.pdf]
